# Influenza and pneumococcus vaccination rates in pediatric dialysis patients in Europe: recommendations vs reality A European Pediatric Dialysis Working Group and European Society for Pediatric Nephrology Dialysis Working Group study

**DOI:** 10.3906/sag-2012-26

**Published:** 2021-02-03

**Authors:** Yeşim Özdemir ATİKEL, Sevcan A BAKKALOĞLU, Fabio PAGLIALONGA, Constantinos J STEFANIDIS, Varvara ASKITI, Enrico VIDAL, Gema ARICETA, Engin MELEK, Enrico VERRINA, Nikoleta PRINTZA, Karel VONDRAK, Aleksandra ZUROWSKA, Ilona ZAGOZDZON, Mesiha EKİM, Elif Nursel ÖZMERT, Stephanie DUFEK, Augustina JANKAUSKIENE, Claus Peter SCHMITT, Eszter LÉVAI, Johan Vande WALLE, Nur CANPOLAT, Tuula HOLTTA, Michel FISCHBACH, Ariane ZALOSZYC, Guenter KLAUS, Christoph AUFRICHT, Rukshana SHROFF, Alberto EDEFONTI

**Affiliations:** 1 Department of Pediatric Nephrology, Faculty of Medicine, Gazi University, Ankara, Turkey; 2 Fondazione IRCCS Ca’ Granda Ospedale Maggiore Policlinico, Milan, Italy; 3 Department of Pediatric Nephrology, Mitera Children’s Hospital, Athens, Greece; 4 Department of Pediatric Nephrology, A. and P. Kyriakou Children’s Hospital, Athens, Greece; 5 Department of Pediatric Nephrology, Faculty of Medicine, Padova University, Padova, Italy; 6 Department of Pediatric Nephrology, University Hospital Vall d’ Hebron, Barcelona, Spain; 7 Department of Pediatric Nephrology, Faculty of Medicine, Çukurova University, Adana, Turkey; 8 Dialysis Unit, Department of Pediatrics, IRCCS Istituto Giannina Gaslini, Genoa, Italy; 9 1st Pediatric Department, Faculty of Medicine, Aristotle University of Thessaloniki, Thessaloníki, Greece; 10 Department of Pediatric Nephrology, Faculty of Medicine, University Hospital Motol, Prague, Czech Republic; 11 Department of Pediatrics Nephrology & Hypertension, Medical University of Gdansk, Gdansk, Poland; 12 Department of Pediatric Nephrology, Faculty of Medicine, Ankara University, Ankara, Turkey; 13 Department of Social Pediatrics, Faculty of Medicine, Hacettepe University, Ankara, Turkey; 14 Department of Pediatric Nephrology, Great Ormond Street Hospital for Children, London, UK; 15 Pediatric Center, Institute of Clinical Medicine, Vilnius University, Vilnius, Lithuania; 16 Pediatric Nephrology, Center for Pediatric and Adolescent Medicine, Heidelberg, Germany; 17 Department of Pediatric Nephrology, Utoped, Universitair Ziekenhuis Gent, Ghent, Belgium; 18 Department of Pediatric Nephrology, Cerrahpaşa Medical School, İstanbul, Turkey; 19 Department of Pediatric Nephrology, Faculty of Medicine, University of Helsinki, Helsinki, Finland; 20 Department of Pediatric Nephrology, Hopital de Hautepierre, Strasbourg, France; 21 Department of Pediatric Nephrology, KfH Children’s Kidney Center, Marburg, Germany; 22 Department of Pediatric Nephrology, Medical University of Vienna, Vienna, Austria

**Keywords:** Dialysis, immunization, influenza vaccine, pneumococcal vaccine, vaccination

## Abstract

**Background/aim:**

Children on dialysis are under increased risk of influenza and invasive pneumococcal disease. Although vaccination against these microorganisms are recommended in dialysis patients and despite the fact that these vaccines can reduce disease burden and rates of hospitalization due to infection, vaccination rates are below expected and desired. We aimed to evaluate influenza and pneumococcal vaccination and infection rates in European pediatric dialysis centers.

**Materials and methods:**

In 16 centers from 11 countries, 357 pediatric dialysis patients were evaluated retrospectively during 1 year of observation period between 01.01.2014 and 01.01.2015.

**Results:**

In all centers, vaccination policy included immunization of dialysis patients with inactive influenza vaccine and pneumococcal conjugate vaccine (PCV). Fifty percent of the centers recommended pneumococcal polysaccharide vaccine following routine PCV series. A significantly higher pneumococcal vaccination rate (43.9%) was seen in peritoneal dialysis (PD) patients compared to those on hemodialysis (HD) (32.9%) (p = 0.035), while the rates for influenza were similar (42.4% and 46.1% respectively, p = 0.496). Among all dialysis patients, 2.2% (n = 8) developed pneumonia and 6.4% (n = 23) was infected by Influenza. Pneumococcic pneumonia rate was 5% for 140 patients who received antipneumococcal vaccine, while only one pneumonia episode was recorded out of 217 unvaccinated patients (p = 0.007). The influenza virus infection rates were similar for patients vaccinated and nonvaccinated (7 % and 6 %, respectively).

**Conclusions:**

Although influenza and pneumococcal vaccines are highly recommended in pediatric dialysis patients, vaccination rates were lower than expected. Pneumococcal vaccination rates were higher in PD compared to the patients on HD. The rate of children with influenza infection was higher than pneumonia. The efficacy of influenza and pneumococcal vaccines was highlighted by the low infection rates. Higher pneumonia rates in patients vaccinated against pneumococcus compared to unvaccinated ones might be due to coexisting risk factors.

## 1. Introduction

Although, in recent years, widespread vaccination in the general pediatric population has significantly reduced the circulation of vaccine-preventable infections, which indirectly reduces the risk for infections in children with chronic kidney disease (CKD) [1], patients on dialysis and candidates of renal transplantation (RT) still require immunizations not routinely provided to healthy children at all age groups [2] including influenza and pneumococcal polysaccharide vaccines (PPSV) due to disease-related complications [1–4].

Children with CKD and RT are at a high risk of developing invasive pneumococcal disease (IPD) [5]. Since 23-valent pneumococcal vaccine decreases the incidence of IPD [6], PPSV should be advocated in all patients with CKD (adult and pediatric), as early in their disease course as possible [6,7].

The influenza virus is highly contagious and its epidemics lead to increased morbidity and mortality with high complication rates principally among people at risk including patients with CKD receiving immunosuppressive treatment and patients on dialysis [8,9]. Despite potentially impaired antibody responses, adult studies showed that vaccinated patients on dialysis had a significant lower rate of all-cause hospitalization risk and mortality rate related to influenza when compared to the unvaccinated ones [9,10]. Many national immunization advisory groups recommend annual influenza vaccination for patients with end-stage renal disease (ESRD) including those on dialysis [6,11–13].

Despite the benefits and vaccination recommendations, studies in chronic kidney patients, including dialysis and kidney transplant candidates, show that actual vaccination rates seem to be lower than expected and desired [4,14–17]. Since the data about the immunization status against influenza and pneumococcus in children maintained by dialysis is rare, we aimed to evaluate influenza and pneumococcal vaccination and respective infection rates in European pediatric dialysis patients.

## 2. Patients and methods

This was a multinational, multicenter, retrospective study including patients from 16 tertiary pediatric nephrology centers from 11 European countries (Belgium, Czech Republic, France, Germany, Greece [2 centers], Italy [3 centers], Lithuania, Poland, Spain, Turkey [3 centers], and United Kingdom). Influenza (inactive influenza vaccine) and pneumococcal vaccination (pneumococcal conjugate vaccine [PCV] and PPSV) rates in 357 prevalent dialysis patients (205 peritoneal dialysis (PD), 152 hemodialysis (HD)) who were younger than 18 years and under regular follow-up were recorded during the 1 year period between 01.01.2014 and 01.01.2015. When evaluating the influenza vaccine coverage, vaccinations for 2014–2015 influenza season were included. Influenza and pneumonia episodes in dialysis patients during the 1 year study period were also noted. Data were obtained from each center through a questionnaire completed by pediatric nephrologists. Statistical analyses were performed using SPSS version 23 software. The study design was approved by the ethics committee of the coordinating center (Gazi University Non-interventional Clinical Research Ethics Committee, approval number and date: 139/14.12.2015) and we conducted this study in accordance with Good Clinical Practice Guidelines.

## 3. Results

Policies about influenza and pneumococcus vaccinations in the centers participating in the study are summarized in Table 1. In all centers, vaccination policy includes vaccination of dialysis patients against influenza, with double-dose being administered in Spain and UK. Vaccination with PCV which was included in national universal immunization program of all participating countries also recommended for children with CKD. Following routine PCV series in infancy, dialysis patients are recommended to be vaccinated by PPSV in eight centers from seven countries.

Details on vaccination status are given in Table 2. Of 357 prevalent dialysis patients, 39.2% (n = 140; 90 on PD and 50 on HD) were vaccinated against pneumococcus with PCV, while 44% (157: 87 on PD and 70 on HD) against influenza, and the vaccination rates were similar (p = 0.197). In addition, 17.6% of patients (n = 63) were also vaccinated with PPSV. When compared by dialysis modality, higher pneumococcal vaccination rate (43.9 %) was seen in 205 PD patients compared to 152 on HD (32.9%), and this rate was found to be statistically significant (p = 0.035). Of 152 HD patients, 20.4% were vaccinated with PPSV after completion of routine PCV series, while this rate was 15.6% among 205 PD patients (p = 0.241). Patients receiving PPSV after completion of PCV series were older than 2 years. 42.4% of the PD patients and 46.1% of the HD patients were vaccinated against influenza (p = 0.496).

During the study period, it was reported that among all dialysis patients, 8 patients (2.2%) developed pneumonia and 23 (6.4%) was infected by influenza. Among 140 patients vaccinated against pneumococcus, seven (5%) experienced pneumonia, while only one pneumonia episode was recorded out of 217 unvaccinated patients; there was a statistically significant difference (p = 0.007). Three patients had pulmonary comorbidity and one had immune deficiency. PPSV had been applied to only one of the 7 vaccinated patients. All pneumonia episodes required hospitalization and resulted in partial or complete recovery.

In 157 patients vaccinated against influenza, 11 influenza episodes (7%) were recorded, while 12 episodes occurred in the 200 unvaccinated ones (6%); the difference was not statistically significant (p = 0.867). Six of the vaccinated patients were hospitalized for influenza and they recovered without any complications. Two patients had both influenza and pneumonia during the observation period; one of them was a 10-year-old female with no comorbid conditions and had been on HD for 66 months, and the other was a 2-year-old patient on PD for 15 months who had pulmonary dysplasia. The detailed data of vaccinated patients experienced pneumonia and influenza are given in Table 3.

In this multinational study, we evaluated influenza and pneumococcal vaccination recommendations and vaccination coverage among 16 European pediatric nephrology centers.

According to this study, in all participating centers, pediatric dialysis patients were recommended to be vaccinated with inactive influenza vaccine and PCV. Following routine PCV series in infancy in all centers, dialysis patients were recommended to be vaccinated with polysaccharide vaccine in half of the centers.

Although CKD patients have for long been included in the high-risk population groups who should receive antipneumococcal vaccine and should annually be vaccinated against influenza, and these recommendations were fully accepted for pediatric nephrology centers, it was observed that, among 357 prevalent dialysis patients, 39% were vaccinated with PCV and 44% received influenza vaccine. 17.6% of patients were also vaccinated with PPSV. Despite the well-known risks and vaccine recommendations, other studies in CKD patients have also demonstrated that vaccination rates are below expected and desired [4,14–17]. A study analyzing the rate of immunization in 62 children undergoing dialysis and RT between 1987 and 2000 showed that 16 patients (25.8%) either on PD or with nephrotic syndrome were vaccinated with the nonconjugate 23-valent vaccine against *S. pneumoniae* [4]. According to the United States Renal Data System (USRDS) data, between 2005 and 2008, 32% of patients in the 0–19 age group with ESRD had influenza vaccine and 13% had pneumococcal vaccine [14]. Between 2008 and 2011, these rates were reported as 40% and 16%, respectively [15]. Pneumococcal and influenza vaccination rates revealed in our study were higher compared to USRDS 2008–2011 pediatric dialysis vaccination data. In a single-center study from Brazil evaluating the vaccination status in pediatric renal transplant recipients, 10.8% of patients had received antipneumococcal vaccine prior to RT [16]. A recent multicenter retrospective study evaluating vaccination coverage in 254 European (from Germany, Italy, Turkey, and the United Kingdom) children with end-stage kidney disease and those on dialysis prior to RT showed that these rates still remain low: 42% of patients received a complete vaccination schedule against pneumococcal infections and 43.3% of transplant candidates received at least one dose of influenza vaccine [17]. Vaccinations of children with CKD might be overlooked as “minor” problems compared with growth, nutrition, dialysis, and social difficulties [4]. Although, antipneumococcal vaccine rates showed improvement from year to year while influenza vaccine rates remained relatively constant, the higher rates of influenza vaccination compared to pneumococcus in those studies, including the current one, might be due to the impact of annual reminders by healthcare providers or media for seasonal use.

According to the 2013 United States Renal Data System (USRDS) report, pediatric HD patients are more likely to be vaccinated against Influenza than children on PD or those with a functioning kidney transplant [15]. In this study, vaccination rates with PPSV and inactive influenza vaccine were similar for the PD and HD patients; however, PD patients were more likely to be vaccinated with antipneumococcal vaccine with a rate of 43.9% compared to those on HD (32.9%). This may be related to younger age of the PD patients in whom vaccination is pursued more seriously.

## 4. Discussion

In this study, the rate of children on dialysis with influenza infection was found to be higher (6.4%) than pneumonia (2.2%). CKD patients remain particularly vulnerable to invasive pneumococcal infection due to reduced immune protection; in particular, children with nephrotic syndrome and elderly on dialysis are at highest risk [18]. In a study, with a 25.8% rate of PPSV vaccination in 62 patients, only one episode of peritonitis was reported which was caused by *S. pneumoniae* [4]. In the present study, 5% of patients vaccinated against pneumococcus experienced pneumonia while only one pneumonia episode was recorded in the unvaccinated children. Half of the patients who experienced pneumococcic pneumonia had comorbid conditions increasing the risk of pneumonia. It was reported that, PPSV had been applied to only one of the 7 vaccinated patients and four patients who had comorbidity did not receive PPSV. All pneumonia episodes required hospitalization, but there was no death associated with pneumonia. More pneumonia episodes, in vaccinated compared to nonvaccinated patients may be due to comorbid conditions and low rate of PPSV vaccination, or may be more attention had been paid on vaccination in high risk patients with comorbidities. Nonetheless, the low rate of pneumococcal pneumonia in all dialysis patients is a promising finding that may indicate the effectiveness of the vaccine.

Patients with ESRD have lower response to influenza vaccines, but still, the vaccines had significantly lower infection-related hospitalization rates and mortality than those who were not vaccinated [18]. Throughout the study period, influenza infection rates were similar between vaccinated and unvaccinated patients (7% vs 6%). All of the vaccinated patients recovered with complete recovery and only half of them were hospitalized for influenza. These results support the view that the influenza vaccine reduces infection related complications and hospitalization [9,10]. In a previous study reporting a high influenza immunization rate (70.9% of 62 children) and low prevalence of influenza in children undergoing dialysis and RT, none of the patients, including those who were not vaccinated, developed a “flu-like” syndrome requiring hospital admission [4]. The vaccine efficacy may be measured by the absence or low prevalence of disease, but of course, it should be confirmed in large cohort studies. Our study included a higher number of dialysis patients. Although the number of patients vaccinated with influenza was more than those vaccinated with antipneumococcal vaccine, the higher rate of influenza infection and similar rates between the vaccinated and nonvaccinated groups can be explained by the fact that influenza is more contagious and the vaccination rate is low in the whole population.

It is important to prevent influenza and pneumococcal infections in these patients who may experience very serious consequences as a result of their already existing serious chronic diseases and other comorbid conditions. As underlined in a study [4], to increase the vaccination coverage in dialysis patients, vaccinations in these populations should not be overlooked, have to be assessed as a part of the primary work-up, and the renal team should take over the responsibility for the reviewing, monitoring, and administration of the vaccines. The household contacts of these patients should also be vaccinated against Influenza and *S. pneumoniae* [4]. Especially, annual vaccination of household contacts with influenza vaccine may reduce the exposure of dialysis patients to the virus.

## 5. Conclusion

Despite the full recommendation of inactive influenza and routine PCV series for pediatric dialysis patients, and in half of the centers PPSV was recommended, vaccination rates with all three vaccines were suboptimal; nevertheless, vaccination rates against Influenza and *S. pneumoniae* were higher compared to those of the United States. Pneumococcal vaccination rates were higher in PD patients compared to those on HD. The respective infection rates were generally low, while the rate of children on dialysis with influenza infection was higher than pneumonia. Higher pneumonia rates in patients vaccinated against pneumococcus compared to unvaccinated ones may be due to coexisting risk factors. The efficacy of influenza and pneumococcal vaccines was highlighted by the low infection rates, and the importance of influenza vaccine had been shown by the results that half of the infected dialysis patients did not necessitate hospitalization and all showed complete recovery. Strategies should be developed to increase vaccination coverage in this patient group.

## Figures and Tables

**Table 1:** Center policy for influenza and pneumococcal vaccines in CKD and dialysis patients.

Vaccine	Recommended	Not recommended
Inactive annual influenza	All centers	-
PCV	All centers	-
PPSV after completion of PCV	CZ, DE, ES, FR, GR (I, II), TR (I), UK	BE, IT (I–III), LT, PL, TR (II, III)

BE: Belgium, CZ: Czech Republic, DE: Germany, ES: Spain, FR: France, GR: Greece, IT: Italy, LT: Lithuania, PCV: pneumococcal conjugated vaccine, PL: Poland, PPSV: pneumococcal polysaccharide vaccine, TR: Turkey, UK: United Kingdom

**Table 2:** Prevalent dialysis patients and their vaccinations against pneumococcus and influenza.

Country	Prevalent dialysis patients HD/PD (n)	Patients vaccinated with PCV (n) HD/PD	Patients vaccinated with PPSV after completion of PCV (n) HD/PD	Patients vaccinated against influenza (n) HD/PD
BE	9/0	1/0	1/0	1/0
CZ	5/12	1/6	0/0	1/2
FR	6/5	6/5	6/5	2/2
DE	10/27	8/16	1/4	9/18
GR	11/18	6/7	6/7	11/14
IT	31/34	5/20	0/1	19/16
LT	1/5	1/1	0/0	0/0
PO	6/4	2/3	0/0	2/2
ES	8/3	7/2	7/2	8/3
TR	37/76	9/20	6/4	8/9
UK	28/21	4/10	4/9	9/21
Total	152/205	50 /90	31/32	70/87

BE: Belgium, CZ: Czech Republic, DE: Germany, ES: Spain, FR: France, GR: Greece, HD: hemodialysis, IT: Italy, LT: Lithuania, PCV: pneumococcal conjugated vaccine, PD: peritoneal dialysis, PL: Poland, PPSV: pneumococcal polysaccharide vaccine, TR: Turkey, UK: United Kingdom

**Table 3:** Vaccinated patients who developed pneumonia and influenza infection.

Patient	Age (months)	Sex	Dialysis modality	Time on dialysis (months)	Primary renal disease	Comorbidity	Vaccine	Hospitalization
*Pneumonia*								
1	19	Male	PD	15	CAKUT	Pulmonary hypoplasia	PCV13	+
2	21	Female	PD	14	CAKUT	-	PCV13	+
3	23	Male	PD	21	CAKUT	-	PCV13	+
4	25	Male	HD	19	CAKUT	Chronic lung disease	PCV13	+
5	41	Male	HD	36	ARPKD	Pulmonary hypoplasia	PCV13	+
6	121	Female	HD	66	RVT	-	PCV7 + PPSV	+
7	148	Male	PD	80	FSGS	Schimke immuno-osseous dysplasia	PCV7	+
*Influenza*								
1	19	Male	PD	15	CAKUT	Pulmonary hypoplasia	1 dose	+
2	24	Male	HD	7	Congenital nephrotic syndrome	-	1 dose	+
3	30	Male	PD	9	Neonatal cortical necrosis	Severe neurologic impairment	1 dose	+
4	35	Male	PD	28	CAKUT	-	1 dose	−
5	47	Male	HD	17	Unknown	-	1 dose	+
6	93	Female	PD	9	Chronic tubulointerstitial nephritis	-	1 dose	−
7	121	Female	HD	66	RVT	-	1 dose	−
8	150	Male	PD	25	Cystinosis	-	1 dose	−
9	158	Male	HD	63	CAKUT	Autism	1 dose	−
10	172	Male	HD	25	CAKUT	-	1 dose	+
11	207	Male	HD	44	HIV associated nephropathy	-	1 dose	+

ARPKD: autosomal recessive polycystic kidney disease, CAKUT: congenital anomalies of the kidney and urinary tract, FSGS: focal segmental glomerulosclerosis, HD: hemodialysis, PD: peritoneal dialysis, RVT: renal vein thrombosis
